# Percutaneous Vertebroplasty in a Patient with Chronic Back Pain Caused by Multiple Schmorl’s Nodes: A Case Report

**DOI:** 10.3390/medicina59101839

**Published:** 2023-10-16

**Authors:** Hyung-Joon Park, Hyun-Ji Jo, Jaeeun Lee, Sang-Sik Choi, Chung-Hun Lee

**Affiliations:** 1 Department of Anesthesiology and Pain Medicine, Hanyang University Guri Hospital, 153 Gyeongchun Road, Guri 11923, Republic of Korea; bkjoon720@gmail.com; 2 Department of Anesthesiology and Pain Medicine, Korea University Medical Center, Guro Hospital, 148 Gurodong Road, Seoul 08308, Republic of Korea; johji0921@gmail.com (H.-J.J.); kellyont@gmail.com (J.L.); clonidine@empal.com (S.-S.C.)

**Keywords:** Schmorl’s node, back pain, percutaneous vertebroplasty

## Abstract

*Background and Objectives*: Schmorl’s nodes (SNs), formed by the herniation of intervertebral discs into adjacent vertebral bodies, are generally asymptomatic and do not require treatment. However, certain types of SNs can cause intractable back pain. *Case Presentation*: A 63-year-old man presented to our hospital with back pain after a fall 1 month prior. Physical examination revealed back pain that worsened with movement and paraspinal tenderness. Magnetic resonance imaging (MRI) performed immediately after presentation revealed subacute to chronic compression fractures with SNs at the upper endplates of the 11th and 12th thoracic and 1st lumbar vertebrae. Pain (numeric rating scale (NRS), 7–8/10) persisted despite 6 months of conservative treatment and MRI revealed increased signal intensity in T2-weighted images in the regions around the SNs. Based on these findings, an epidural nerve block was performed, and then repeated; however, no significant improvement was observed. Percutaneous vertebroplasty (PVP) was performed at the 11th and 12th thoracic and 1st lumbar vertebrae. Pain levels decreased substantially 1 week after PVP (NRS, 3–4/10). Subsequent treatment with non-steroidal anti-inflammatory drugs (NSAIDs) and steroids for two weeks further reduced pain levels (NRS, 1–2/10), following which steroid use was discontinued and NSAID use became intermittent. At the six-month follow-up, pain levels remained low and the patient reported an improvement in activity levels of 90% or more. *Conclusions*: This case report demonstrates that PVP safely and effectively improved symptoms in a patient with multiple SNs and intractable back pain. Nevertheless, further research, particularly large-scale randomized prospective studies, is necessary to validate the long-term efficacy and safety of this intervention.

## 1. Introduction

Schmorl’s nodes (SNs) were first described in 1927 as the herniation of an intervertebral disc into an adjacent vertebral body through a vertebral endplate [1]. Although the pathophysiological mechanisms underlying the formation of SNs remain controversial, osteonecrosis under the cartilage endplate and avascular disc herniation resulting from the abnormal development of spinal vessels are considered to be major contributing factors [2].

The prevalence of SNs varies from 9% to 76% [1,3,4]. In healthy individuals, SNs are typically asymptomatic and do not require treatment [3,4]; however, certain types of SNs can cause intractable back pain [4]. Typically, a painful SN is indicated by the appearance of an edematous rim of cancellous bone on magnetic resonance imaging (MRI) [5]. Micromovements, inflammation, and pressure on nociceptors within the edematous bone may cause pain [5]. In addition, the presence of multiple SNs is closely associated with lumbar disc disease and low back pain [1,3].

The first-line treatment for symptomatic SNs is conservative management, including analgesics, bed rest, physical therapy, and the use of braces [6]. However, these treatments are ineffective in certain patients. Several second-line treatments have therefore been proposed for the management of low back pain secondary to suspected painful SNs, such as nerve blocks, discoblocks, fusion, and percutaneous vertebroplasty (PVP) [6,7,8,9]. None of these methods have yet been evaluated by well-structured, randomized controlled trials, and as a result their use remains somewhat controversial. Of these methods, PVP is a minimally invasive procedure that has been shown to provide rapid pain relief and stability in patients with osteoporotic vertebral compression fractures [10].

We encountered a patient with severe back pain lasting more than 6 months, despite conservative management and an epidural nerve block, due to chronic compression fractures accompanied by multiple SNs. MRI revealed that the signal intensity on T2-WI increased progressively around SNs. Here, we report a case in which PVP significantly alleviated back pain.

## 2. Case Presentation

This study was approved by the Institutional Review Board of Korea University Medical Center, Guro Hospital, Seoul, Republic of Korea (2023GR0210) on 5 June 2023.

A 63-year-old man visited our hospital with persistent back pain after a fall 1 month before presentation. Six months prior to presentation, the patient received treatment for lower extremity pain caused by L5 nerve root compression but had no other relevant medical history.

**Figure 1:**
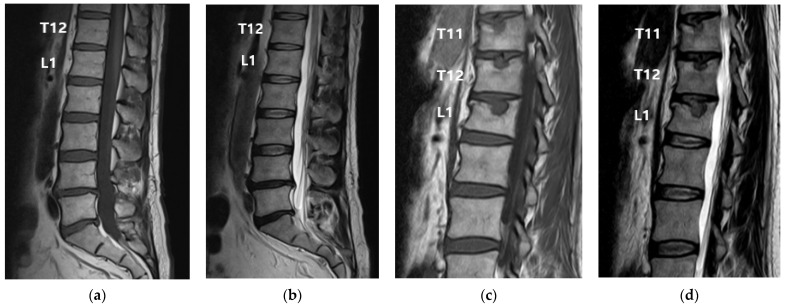
Magnetic resonance imaging (MRI) performed 6 months before, and immediately after, presentation. The patient had experienced a fall 1 month prior to presentation. (**a**) T1-weighted and (**b**) T2-weighted MRI performed 6 months before presentation. No abnormalities were observed in the 12th thoracic or 1st lumbar vertebrae. (**c**) T1-weighted and (**d**) T2-weighted MRI performed immediately after presentation. A new subacute to chronic compression fracture with SNs on the upper endplates of the 11th and 12th thoracic and 1st lumbar vertebrae was observed.

Pain at the time of the visit was mainly in the thoracolumbar junction area without radiating to the legs; the numerical rating scale (NRS) score was 8/10 (0, no pain; 10, maximum amount of pain imaginable). The pain worsened with movement and did not completely disappear when the patient stopped moving. Physical examination revealed spinous process and paraspinal tenderness at the 11th and 12th thoracic and 1st lumbar vertebrae. The results of both straight leg raise tests, Spurling’s test, and Lhermitte’s sign were negative. No blisters or rashes were observed. Osteoporosis with a T-score of −2.6 was observed on the bone density test performed using dual-energy X-ray absorptiometry. Owing to the patient’s age and the findings of osteoporosis, worsening pain during motion, and tenderness, a spinal MRI was performed to identify any fractures or additional causative factors. This MRI, compared with that performed 6 months prior, revealed a new subacute to chronic compression fracture with SNs on the upper endplates of the 11th and 12th thoracic and 1st lumbar vertebrae (Figure 1). There were no other abnormal findings.

Analgesic drugs (tramadol and acetaminophen), physical therapy, and orthoses were administered to alleviate back pain. Initially, the patient experienced pain relief (NRS, 4–5/10); however, the response to treatment declined over time and the pain increased (NRS, 6–7/10). Two months after presentation, the analgesic drug was changed to oxycodone and non-steroidal anti-inflammatory drugs (NSAIDs) were added to the treatment regimen; however, there was no improvement in the symptoms (NRS, 6–7/10). After 5 months of conservative treatment, the patient experienced worsened pain (NRS, 7–8/10); therefore, lumbar MRI and whole-body bone scintigraphy were performed to identify additional causes of back pain.

**Figure 2:**
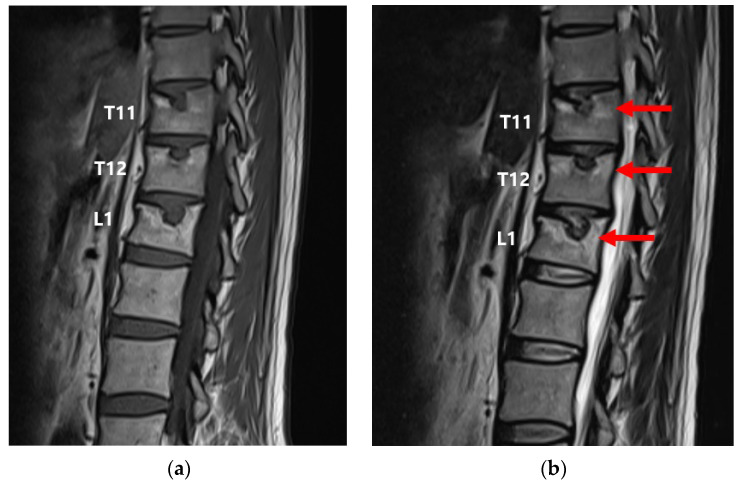
(**a**) T1-weighted and (**b**) T2-weighted MRI performed 5 months after presentation, 6 months after a fall. Signal intensity was increased (red arrows) in the upper endplate regions of the 11th and 12th thoracic and 1st lumbar vertebrae compared to that seen in the MRI performed immediately after presentation. An increase in the size of the SNs was also observed.

This MRI revealed a greater signal intensity on T2-WI in the upper endplate regions of the 11th and 12th thoracic and 1st lumbar vertebrae, and an increase in the size of the SNs, compared with the MRI performed immediately after presentation (Figure 2).

**Figure 3:**
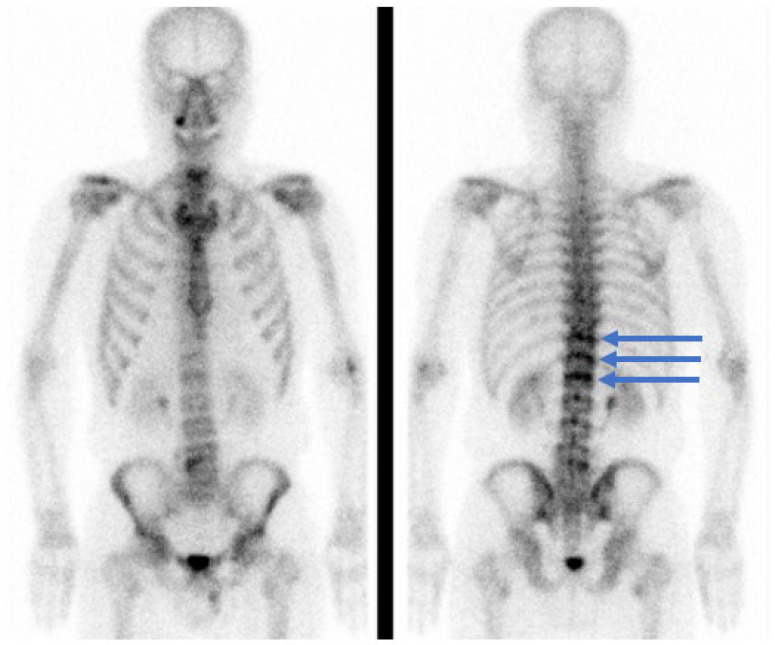
Whole-body bone scintigraphy performed 5 months after presentation, 6 months after a fall. Radionuclide uptake was increased (blue arrows) in the upper endplate regions of the 11th and 12th thoracic and 1st lumbar vertebrae.

Whole-body bone scintigraphy revealed increased radionuclide uptake in the upper endplate regions of the 11th and 12th thoracic and 1st lumbar vertebrae (Figure 3).

**Figure 4:**
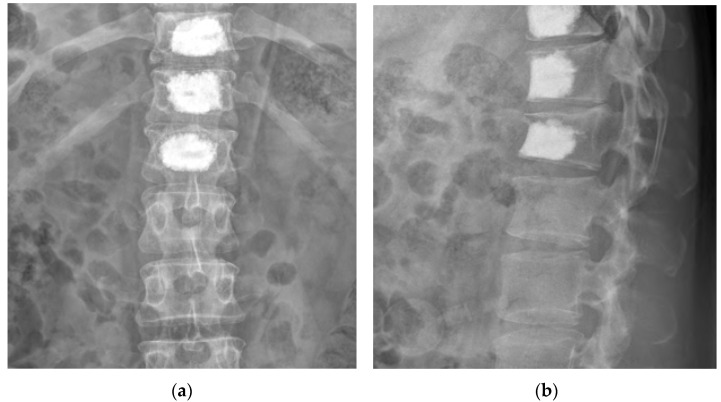
Thoracolumbar radiography after percutaneous vertebroplasty of the 11th and 12th thoracic and 1st lumbar vertebrae. (**a**) Anteroposterior and (**b**) lateral views.

Based on physical examination, MRI, and bone scintigraphy findings, we determined that the multiple SNs were the major cause of pain, and two interlaminar nerve blocks were performed in the epidural space of the 12th thoracic vertebra and the 1st lumbar vertebra. However, this resulted in minimal pain relief (NRS, 7/10), and the persistent pain experienced by the patient resulted in a gradual reduction in activity levels. Therefore, following consultation with the patient, PVP was performed on the 11th and 12th thoracic and 1st lumbar vertebrae (Figure 4).

PVP was performed according to established methods. To correct kyphosis, the patient was placed on a table in a prone position with a pad placed under the abdomen. After checking vital signs, a sterile dressing was applied to the treatment area, and local anesthesia was induced using 1% lidocaine. Using a fluoroscopic guide, a needle was advanced through the pedicle into the target vertebral body using a unilateral transpedicular approach. After inserting the guidewire, a cannula was inserted, such that its end was positioned outside the posterior third of the vertebral body. Subsequently, bone cement (polymethylmethacrylate) was injected through the cannula until it reached the superior endplate and the anterior and posterior cortical margins with the SN, as observed by fluoroscopy; the procedure was terminated once sufficient bone cement was injected. PVP was performed on the 11th and 12th thoracic and 1st lumbar vertebrae using this method.

The patient reported an NRS score of 1/10 on the day after PVP; this increased slightly to 3–4/10 a week after PVP. NSAIDs and steroids were prescribed for 2 weeks for the treatment of residual pain. Subsequently, steroid treatment was discontinued, and treatment with NSAIDs became intermittent. At outpatient follow-up visits one, three, and six months after PVP, the patient reported that only a slightly stiff feeling remained; pain was significantly improved compared with that before the procedure (NRS, 1–2/10). The patient also reported an improvement in activity levels of 90% or more following PVP.

## 3. Discussion

In the case reported here, PVP was an effective treatment for back pain caused by chronic vertebral compression fractures accompanied by multiple SNs that did not respond to conservative treatment.

Symptomatic SNs may develop due to an inappropriate immune response triggered by the prolapse of the nucleus pulposus into the spine, potentially resulting in an imbalance in bone deposition and resorption and subsequent bone loss [1]. Bone loss exacerbates the condition by increasing the susceptibility of the affected vertebrae to herniation of the disc material, and symptoms appear due to compression of nearby nociceptive nerve endings, as SNs become surrounded by calcified material [1].

According to a study by Takahashi et al., on patients with SNs, the risk of low back pain increased if the SNs exhibited low signal intensity on T1-WI and high-intensity signals on T2-WI, indicating acute edema of the bone marrow [5]. In addition, according to a long-term follow-up study of patients with SNs by Wu et al., approximately 26% of SNs increased in size and 13% demonstrated significantly increased signal intensity on T2-weighted MRI [11]. This SN progression was associated with vertebral compression fractures and pain [11]. Endplates surrounding the SNs with high signal intensity in the fat suppression sequence of spinal MRI exhibit acute bone marrow edema and can cause severe back pain, similar to acute vertebral compression fractures. In the present case, the high-intensity signal surrounding the SNs, and the size of the SNs, were increased on T2-WI performed 6 months after the fall compared with that observed 1 month after the fall. Based on these findings, we identified the SNs as the cause of intractable back pain. In addition, the patient had SNs at the level of three vertebrae, namely the 11th and 12th thoracic and 1st lumbar vertebrae. Kyere et al., reported that multiple SNs are closely associated with lower back pain [1]. Bone scintigraphy performed 6 months after the fall demonstrated increased radionuclide uptake in the upper endplate regions of the 11th and 12th thoracic and 1st lumbar vertebrae; this is associated with recent fractures and can predict pain relief after PVP [12,13]. Therefore, we concluded that the SNs were the main cause of intractable back pain in the patient.

First, based on previous studies showing that nerve block is effective in patients with symptomatic SNs, a block was performed at the epidural level of the 11th and 12th thoracic and 1st lumbar vertebrae [8,14]. However, back pain persisted even after repeated nerve block administration. Various treatment methods were considered. Discoblock administration has been reported to be effective for the treatment of symptomatic SNs [9]. However, discoblock is not typically performed at our center owing to the risk of infection. Additionally, the patient was desirous of a more powerful and reliable treatment method after two unsuccessful epidural nerve blocks. Therefore, after consultation with the patient and his family, we decided to perform PVP. Symptomatic SNs, such as vertebral compression fractures, cause pain by activating the pain receptors located in the annulus fibrosus and periosteum of the vertebral body [15,16]. Therefore, PVP, which is an effective treatment for vertebral compression fractures, was also effective for the treatment of symptomatic SNs in this case, with the patient experiencing significant pain relief after PVP.

However, the patient reported increased pain 1 week after PVP. We considered various possible explanations for this. The efficacy of PVP treatment lies in artificially accelerating local sclerosis. Inadequate cement distribution may prevent sclerotic bone from surrounding the SN and has been reported to have a negative impact on clinical outcomes such as persistent pain [17]. However, in this case, cement was injected until it reached the upper endplate where the SN is located, and the front and rear cortical edges (cement injection volume: T11, 6 mL; T12, 6 mL; L1, 6.5 mL) (Figure 4). Therefore, we considered other potential causes.

Inflammatory changes and cellular infiltration can be induced by the contact between intracavernous disc components and vertebral bone marrow in the SN [18]. In other words, when the nucleus pulposus, accompanied by abnormal blood vessels, is herniated through the weakened endplate in the SNs, and the vulgar nucleus enters the vascular tissue, the immune system recognizes them as foreign and initiates an immune response, leading to inflammatory cell migration and neovascularization [1,18]. The resulting inflammation, edema, and cytokine secretion can aggravate pain [1]. Reports have described a reduction in C-reactive protein and pain levels following treatment with NSAIDs in some patients with SNs [19,20]. Therefore, in this case, NSAIDs and a strong anti-inflammatory steroid were administered for 2 weeks, following which the patient’s NRS score decreased to 1/10. This was despite the lack of response to NSAID treatment before PVP. It may be that, after PVP relieved the pain caused by mechanical stress on the nociceptors located in the edematous ring around the SNs, NSAIDs provided pain relief from the residual inflammation [18].

Few studies have reported the effectiveness of PVP in patients with symptomatic SNs [16,21,22]. He et al., performed PVP in 11 patients with SNs who did not respond to conservative treatment and reported immediate and long-term pain relief [21]. In addition, Zhi-Yong et al., retrospectively studied percutaneous kyphoplasty in 32 patients with vertebral compression fractures accompanied by SNs and reported stabilization of vertebral body height and functional improvement; these effects were maintained throughout the five-year follow-up period [22]. Several retrospective studies have reported that PVP has a significant effect on patients with SNs; however, the efficacy of this treatment has not been definitively proven [16]. Therefore, additional reports and large-scale randomized prospective studies are necessary. Owing to the retrospective nature of case reports, objective indicators other than the patient’s pain level, such as activity improvement, could not be applied in this study. Therefore, additional outcome measures should be incorporated into future large-scale randomized prospective studies to evaluate the effects of PVP and any adverse events.

## 4. Conclusions

Currently, there is no established method of pain management in patients with symptomatic SNs. However, this case report demonstrates that PVP safely and effectively improved symptoms in a patient with multiple SNs and intractable back pain. Further research, particularly large-scale randomized prospective studies, is necessary to validate the long-term efficacy and safety of this intervention.

## Data Availability

All relevant data are contained within the manuscript.

## References

[1] Kyere K.A., Than K.D., Wang A.C., Rahman S.U., Valdivia-Valdivia J.M., La Marca F., Park P. (2012). Schmorl’s nodes. Eur. Spine J..

[2] Peng B., Wu W., Hou S., Shang W., Wang X., Yang Y. (2003). The pathogenesis of Schmorl’s nodes. J. Bone Joint. Surg Br..

[3] Williams F.M., Manek N.J., Sambrook P.N., Spector T.D., Macgregor A.J. (2007). Schmorl’s nodes: Common, highly heritable, and related to lumbar disc disease. Arthritis Rheum..

[4] Zhang Y., Yin P., Yang J., Hai Y. (2020). Percutaneous vertebroplasty (PVP) to treat a specialized type of endplate fractures around the Schmorl’s node: A prospective study of 65 patients. J. Orthop. Surg. Res..

[5] Takahashi K., Miyazaki T., Ohnari H., Takino T., Tomita K. (1995). Schmorl’s nodes and low-back pain. Analysis of magnetic resonance imaging findings in symptomatic and asymptomatic individuals. Eur. Spine J..

[6] Masala S., Pipitone V., Tomassini M., Massari F., Romagnoli A., Simonetti G. (2006). Percutaneous vertebroplasty in painful schmorl nodes. Cardiovasc. Intervent. Radiol..

[7] Hasegawa K., Ogose A., Morita T., Hirata Y. (2004). Painful Schmorl’s node treated by lumbar interbody fusion. Spinal Cord.

[8] Jang J.S., Kwon H.K., Lee J.J., Hwang S.M., Lim S.Y. (2010). Rami communicans nerve block for the treatment of symptomatic Schmorl’s nodes—A case report. Korean J. Pain.

[9] Liu J., Hao L., Zhang X., Shan Z., Li S., Fan S., Zhao F. (2018). Painful Schmorl’s nodes treated by discography and discoblock. Eur. Spine J..

[10] Phillips F.M. (2003). Minimally invasive treatments of osteoporotic vertebral compression fractures. Spine.

[11] Wu H.T., Morrison W.B., Schweitzer M.E. (2006). Edematous Schmorl’s nodes on thoracolumbar MR imaging: Characteristic patterns and changes over time. Skeletal Radiol..

[12] Maynard A.S., Jensen M.E., Schweickert P.A., Marx W.F., Short J.G., Kallmes D.F. (2000). Value of bone scan imaging in predicting pain relief from percutaneous vertebroplasty in osteoporotic vertebral fractures. AJNR Am. J. Neuroradiol..

[13] Jordan E., Choe D., Miller T., Chamarthy M., Brook A., Freeman L.M. (2010). Utility of bone scintigraphy to determine the appropriate vertebral augmentation levels. Clin. Nucl. Med..

[14] Kim S., Jang S. (2018). Radicular pain caused by Schmorl’s node: A case report. Braz. J. Anesthesiol..

[15] Zhang N., Li F.C., Huang Y.J., Teng C., Chen W.S. (2010). Possible key role of the immune system in Schmorl’s nodes. Med. Hypotheses.

[16] Amoretti N., Guinebert S., Kastler A., Torre F., Andreani O., Foti P., Cornelis F., Theumann N., Hauger O. (2019). Symptomatic Schmorl’s nodes: Role of percutaneous vertebroplasty. Open study on 52 patients. Neuroradiology.

[17] Cai K., Jiang G., Lu B., Zhang K., Luo K. (2023). Bone cement distribution may significantly affect the efficacy of percutaneous vertebroplasty in treating symptomatic Schmorl’s nodes. BMC Musculoskelet. Disord..

[18] Mattei T.A., Rehman A.A. (2014). Schmorl’s nodes: Current pathophysiological, diagnostic, and therapeutic paradigms. Neurosurg. Rev..

[19] Hershkovich O., Koch J.E., Grevitt M.P. (2020). Schmorl Node-A Cause of Acute Thoracic Pain: A Case Report and Pathophysiological Mechanism. Int. J. Spine Surg..

[20] Abu-Ghanem S., Ohana N., Abu-Ghanem Y., Kittani M., Shelef I. (2013). Acute Schmorl node in the dorsal spine: An unusual cause of a sudden onset of severe back pain in a young female. Asian Spine J..

[21] He S.C., Zhong B.Y., Zhu H.D., Fang W., Chen L., Guo J.H., Deng G., Teng G.J. (2017). Percutaneous Vertebroplasty for Symptomatic Schmorl’s Nodes: 11 Cases with Long-term Follow-up and a Literature Review. Pain Physician.

[22] Zhi-Yong S., Huan Z., Feng L., Nan-Ning L., Xiao-Yu Z., Bin P., Jun L., Zhong-Lai Q., Zhi-Ming Z., Hui-Lin Y. (2017). A Retrospective Study of Percutaneous Balloon Kyphoplasty for the Treatment of Symptomatic Schmorl’s Nodes: 5-Year Results. Med. Sci. Monit..

